# Impact of Oral Herbal Supplementation on Human Skin Aging: A Systematic Review of Clinical Trials

**DOI:** 10.1111/jocd.71094

**Published:** 2026-07-27

**Authors:** Gisela Meydilin Sanjaya, Brilian Veda Kartika Putri, Hany Anneke, Nayla Majeda Alfarafisa, Astrid Feinisa Khairani, Nur Atik

**Affiliations:** ^1^ Graduate School of Master Program in Anti Aging and Aesthetic Medicine, Faculty of Medicine Universitas Padjadjaran West Java Indonesia; ^2^ Department of Biomedical Sciences, Faculty of Medicine Universitas Padjadjaran West Java Indonesia

**Keywords:** antioxidants, clinical trial, herbal supplement, human skin aging, oral intervention

## Abstract

**Background:**

Skin aging, characterized by wrinkles, decreased elasticity, and dryness, is both a public health and esthetic issue, affecting most adults and leading to lowered quality of life and higher healthcare costs. While topical treatments are common, systemic approaches are gaining interest for their potential to enhance skin hydration, elasticity, and barrier function. Nonetheless, the effects of oral herbal supplements remain less examined.

**Aim:**

This systematic review evaluated the effectiveness of oral herbal supplements in reducing skin aging in humans, focusing on elasticity, hydration, wrinkle depth, and antioxidant activity.

**Methods:**

Randomized controlled trials (RCTs) were identified through structured database searches (March–August 2025). Twelve RCTs published between 2020 and 2025 met the inclusion criteria, involving adults who received oral herbal supplements versus placebo or standard care. Outcomes measured included skin elasticity, hydration, wrinkle depth, smoothness, antioxidant capacity, and inflammatory markers. Quality assessment using the Cochrane RoB 2 tool indicated that all 12 RCTs were judged as having *some concerns* of bias.

**Results:**

Interventions included *Glycine max* , bilberry, cranberry, Hydrangea serrata, Pycnogenol, and others. Most studies reported significant improvements in dermatological outcomes. Proposed mechanisms involve antioxidant activity, anti‐inflammatory effects, collagen synthesis, barrier restoration, photoprotection, DNA repair, and microbiome modulation.

**Conclusion:**

In conclusion, oral herbal supplements show promising potential in improving skin aging outcomes by targeting multiple biological pathways. Further research with larger populations, longer durations, and biomarker‐based endpoints is needed to confirm efficacy and support standardized use in dermatology and esthetic medicine.

## 1 Introduction

Human skin aging, marked by wrinkles, reduced elasticity, and dryness, is a major global public health and esthetic concern. By age 50, it affects over 80% of adults, lowering quality of life and increasing healthcare costs. Both intrinsic and extrinsic factors, such as oxidative stress and ultraviolet (UV) exposure, accelerate these changes by driving collagen breakdown and inflammation [1].

Growing consumer interest in natural cosmetic ingredients has accelerated innovation in dermatology, with a growing preference for plant‐based formulations perceived as safer and more sustainable. This shift reflects accumulating evidence that dietary antioxidants derived from plants can benefit both systemic and cutaneous health. Recent investigations have identified bioactive phytochemicals with antioxidant, antimicrobial, and anti‐aging properties [2, 3, 4].

While topical treatments are common, oral supplements are gaining popularity for skin health. Collagen, ceramides, hyaluronic acid, and plant antioxidants improve elasticity, hydration, and barrier function. Meta‐analyses show collagen boosts dermal density, while fruit extracts reduce water loss. Herbal supplements rich in flavonols and polyphenols also enhance elasticity and photoprotection, making them a promising noninvasive option for photoaging [5, 6].

Despite growing interest, existing literature shows notable limitations: many trials are small, short‐term (8–24 weeks), and focus narrowly on single compounds (e.g., collagen or fruit extracts), with minimal comparative assessment of herbal formulations. Reviews mainly discuss plant‐derived supplements in topical use, leaving oral herbal supplements less explored [2]. Although systematic reviews confirm moisturizing benefits from oral nutraceuticals, strong clinical data on their effectiveness in reducing wrinkle depth, improving elasticity, or offering photoprotection remain lacking [7].

Another challenge is that many nutraceuticals combine multiple active compounds, making it difficult to determine which ingredient drives the observed effects and complicating safety evaluations [5]. In contrast, trials using herbal supplements without additional active compounds allow clearer interpretation of results and minimize confounding from ingredient interactions. Therefore, this review focuses solely on oral herbal interventions without added actives, allowing more reliable conclusions on efficacy and safety. Notably, no comprehensive review has yet evaluated a wide range of such herbal supplements in randomized clinical trials.

A recent bibliometric analysis demonstrates rapid growth in skin aging research, with Scopus‐indexed clinical trials increasing from seven in 2005 to 93 in 2024, reflecting heightened global interest in anti‐aging interventions and supporting the focus on recent, evidence‐based studies. To ensure methodological relevance, contemporary trials incorporate advanced skin assessment technologies, including Cutometer for elasticity, Corneometer for hydration, Tewameter for Transepidermal water loss (TEWL), as well as ultrasound imaging, pH meters, Sebumeter, Mexameter, and AI‐based skin analysis [8, 9, 10].

This systematic review addresses the research gap by analyzing clinical trials on oral herbal supplements for skin aging, focusing on hydration, elasticity, wrinkle reduction, and photoprotection. By integrating evidence from diverse herbal interventions, it aims to evaluate their effectiveness and safety in improving visible signs of skin aging.

## 2 Materials and Methods

This systematic review follows the PRISMA (Preferred Reporting Items for Systematic Reviews and Meta‐Analyses) guidelines as seen on Table 1. The study aims to examine the scientific literature on the effectiveness of oral herbal supplements in reducing signs of human skin aging and to assess whether clinical outcomes such as skin hydration, wrinkle reduction, elasticity, or pigmentation improve with intervention.

**TABLE 1:** PRISMA checklist.

Section/topic	PRISMA Item	Checklist item description	Location in manuscript
Title	1	Identify the report as a systematic review.	Title page
Abstract	2	Structured summary including background, objectives, methods (eligibility criteria, information sources, risk of bias, synthesis), results, limitations, conclusions, and registration.	Abstract
Introduction	3	Rationale for the review.	Introduction
	4	State the review questions and objectives.	Introduction (final paragraph)
Methods	5	Specify inclusion and exclusion criteria, including study design, participants, interventions, comparators, and outcomes.	Methods
	6	Describe all information sources (databases, search dates).	Methods
	7	Present complete search strategy for all databases.	Methods
	8	Describe the study selection process (screening, eligibility, inclusion).	Methods
	9	Describe the data collection process and extraction methods.	Methods
	10	List and define all variables sought (PICO elements, outcomes).	Methods
	11	Describe methods for assessing risk of bias in included studies.	Methods
	12	Specify effect measures used for outcomes (if applicable).	Methods and Introduction
	13	Describe methods used for synthesis, including reasons for no meta‐analysis.	Methods and Discussion
	14	Describe processes for reporting bias assessment.	Methods
	15	Describe certainty or confidence assessment (e.g., GRADE), if performed.	Not performed; stated in Discussion
Results	16	Provide a PRISMA flow diagram and numbers of included/excluded studies.	Methods and Results—Study Selection; Figure (PRISMA Diagram and table)
	17	Present characteristics of included studies.	Results
	18	Present risk of bias assessments for each included study.	Results
	19	Present results of individual studies.	Results
	20	Present results of synthesis (narrative).	Results
	21	Report results of any assessments of reporting bias.	Discussion
	22	Report certainty/quality of evidence if performed.	Not applicable (narrative review)
Discussion	23	Provide a general interpretation of results in the context of other evidence.	Discussion
	24	Discuss limitations of included studies.	Discussion
	25	Discuss limitations of the review process.	Discussion
	26	Provide implications for practice, policy, or future research.	Discussion
Other information	27	Registration information; indicate review protocol status.	Methods
	28	Indicate sources of support/funding.	Funding Statement
	29	Declare competing interests.	Conflicts of Interest
	30	Describe availability of data, materials, supporting information files.	Data Availability; Supporting Information

The research was designed as a systematic review. The population of interest includes adult individuals aged 20 years or older who exhibit visible signs of skin aging, such as wrinkles, fine lines, decreased elasticity, or uneven pigmentation. The intervention assessed was per oral herbal supplementation, compared to placebo or standard treatments. The outcome evaluated was clinical improvement in skin aging parameters. The review includes only clinical trials conducted on humans, published in English between 2020 and 2025, and published in an open‐access journal.

**FIGURE 1:**
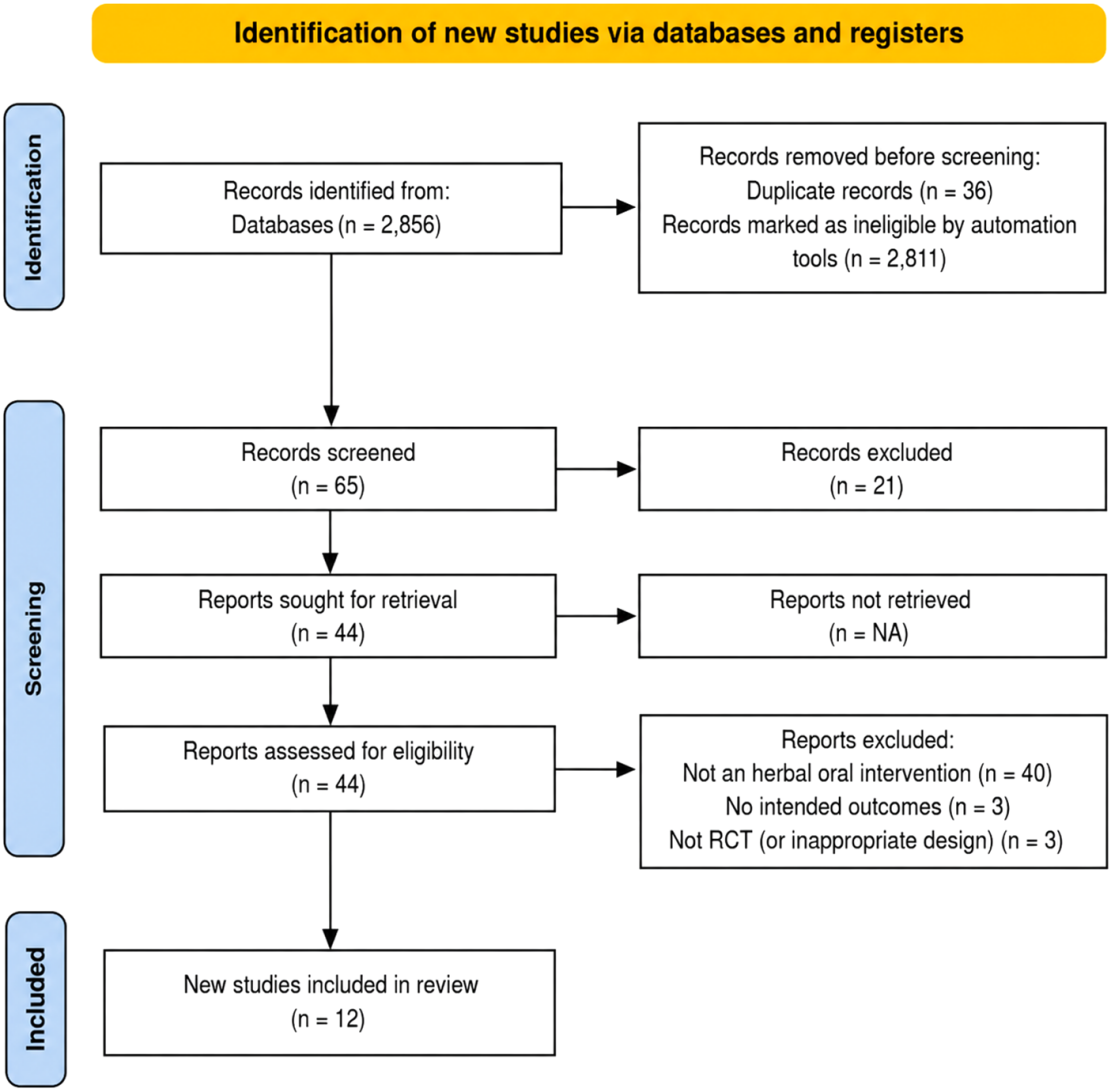
Flowchart of selection process.

A study selection flowchart and full‐text exclusion table (Figure 1 and Table 2) were created using three health databases: PubMed, ScienceDirect, and Cochrane. The Boolean operators (AND, OR, and NOT) were used, and the intersection with the following keywords is “oral” OR “peroral” OR “supplement” AND “herbal” AND “skin aging” OR “skin ageing” AND “human” AND “clinical trial.”

**TABLE 2:** Excluded from full text screening.

No.	First author	Year	Title	Reason for exclusion
1	Caverzan J.	2021	A new phytocosmetic preparation from Thymus vulgaris stimulates adipogenesis and controls skin aging process: In vitro studies and topical effects in a double‐blind placebo‐controlled clinical trial.	Not a herbal oral intervention (e.g., collagen/HA/animal‐derived or synthetic ingredients)
2	Tsuchiya Y.	2020	Safety and efficacy of oral intake of ceramide‐containing acetic acid bacteria for improving the stratum corneum hydration: A randomized, double‐blind, placebo‐controlled study over 12 weeks.	Not a herbal oral intervention (e.g., collagen/HA/animal‐derived or synthetic ingredients)
3	Choi S.Y.	2020	Effects of hyaluronic acid injected using the mesogun injector with stamp‐type microneedle on skin hydration.	Not a herbal oral intervention (e.g., collagen/HA/animal‐derived or synthetic ingredients)
4	Evans M.	2021	A randomized, triple‐blind, placebo‐controlled, parallel study to evaluate the efficacy of a freshwater marine collagen on skin wrinkles and elasticity.	Not a herbal oral intervention (e.g., collagen/HA/animal‐derived or synthetic ingredients)
5	Lin P.	2021	Collagen formula with Djulis for improvement of skin hydration, brightness, texture, crow's feet, and collagen content: A double‐blind, randomized, placebo‐controlled trial.	Not a herbal oral intervention (e.g., collagen/HA/animal‐derived or synthetic ingredients)
6	Maia Campos	2021	Oral supplementation with hydrolyzed fish cartilage improves the morphological and structural characteristics of the skin: A double‐blind, placebo‐controlled clinical study.	Not a herbal oral intervention (e.g., collagen/HA/animal‐derived or synthetic ingredients)
7	Michelotti A.	2021	Oral intake of a new full‐spectrum hyaluronan improves skin profilometry and ageing: A randomized, double‐blind, placebo‐controlled clinical trial.	Not a herbal oral intervention (e.g., collagen/HA/animal‐derived or synthetic ingredients)
8	Piyavatin P.	2021	Synbiotics supplement is effective for Melasma improvement.	Not a herbal oral intervention (e.g., collagen/HA/animal‐derived or synthetic ingredients)
9	Rybak I	2021	Prospective randomized controlled trial on the effects of almonds on facial wrinkles and pigmentation.	Not a herbal oral intervention (e.g., collagen/HA/animal‐derived or synthetic ingredients)
10	Sangsuwan W	2021	Four‐weeks daily intake of oral collagen hydrolysate results in improved skin elasticity, especially in sun‐exposed areas: A randomized, double‐blind, placebo‐controlled trial.	Not a herbal oral intervention (e.g., collagen/HA/animal‐derived or synthetic ingredients)
11	Tsuchiya Y.	2021	Safety evaluation of the excessive intake of ceramide‐containing acetic acid bacteria—a randomized, double‐blind, placebo‐controlled study over a 4‐week period.	Not a herbal oral intervention (e.g., collagen/HA/animal‐derived or synthetic ingredients)
12	Yoshikata R.	2021	Effects of an equol‐containing supplement on advanced glycation end products, visceral fat and climacteric symptoms in postmenopausal women: A randomized controlled trial.	No instrumental skin aging outcome; does not meet predefined outcome criteria
13	Zanini D.	2021	The effects of 6‐month hydrogen‐rich water intake on molecular and phenotypic biomarkers of aging in older adults aged 70 years and over: A randomized controlled pilot trial.	Not a herbal oral intervention (e.g., collagen/HA/animal‐derived or synthetic ingredients)
14	Chan L.P.	2022	Fermented pomegranate extracts protect against oxidative stress and aging of skin.	Not a herbal oral intervention (e.g., collagen/HA/animal‐derived or synthetic ingredients)
15	Han H.S.	2022	Safety and efficacy of high‐intensity focused ultrasound for treatment of periorbital, perioral, and neck wrinkles: Prospective open single‐center single‐arm confirmatory clinical trial.	Not a herbal oral intervention (e.g., collagen/HA/animal‐derived or synthetic ingredients)
16	Kim J.	2022	Oral supplementation of low‐molecular‐weight collagen peptides reduces skin wrinkles and improves biophysical properties of skin: A randomized, double‐blinded, placebo‐controlled study.	Not a herbal oral intervention (e.g., collagen/HA/animal‐derived or synthetic ingredients)
17	Liu C.	2022	The potential of *Streptococcus thermophiles* (TCI633) in the anti‐aging.	Not a herbal oral intervention (e.g., collagen/HA/animal‐derived or synthetic ingredients)
18	Shariff R.	2022	Superior even skin tone and anti‐ageing benefit of a combination of 4‐hexylresorcinol and niacinamide.	Not a herbal oral intervention (e.g., collagen/HA/animal‐derived or synthetic ingredients)
19	Tseng Y.P.	2022	Coffee pulp supplement affects antioxidant status and favors anti‐aging of skin in healthy subjects.	Not a herbal oral intervention (e.g., collagen/HA/animal‐derived or synthetic ingredients)
20	Xie Y.	2022	A new product of multi‐plant extracts improved skin photoaging: An oral intake in vivo study.	Multiple plant extracts
21	Lee M.	2023	Oral intake of collagen peptide NS improves hydration, elasticity, desquamation, and wrinkling in human skin: A randomized, double‐blinded, placebo‐controlled study.	Not a herbal oral intervention (e.g., collagen/HA/animal‐derived or synthetic ingredients)
22	Na G.H.	2023	Skin anti‐aging efficacy of enzyme‐treated supercritical caviar extract: A randomized, double‐blind, placebo‐controlled clinical trial.	Not a herbal oral intervention (e.g., collagen/HA/animal‐derived or synthetic ingredients)
23	Rizzo J.	2023	Soy protein containing isoflavones improves facial signs of photoaging and skin hydration in postmenopausal women: Results of a prospective randomized double‐blind controlled trial.	Not a herbal oral intervention (e.g., collagen/HA/animal‐derived or synthetic ingredients)
24	Shen Shuzhan	2023	Dietary supplementation of n‐3 PUFAs ameliorates LL37‐induced rosacea‐like skin inflammation via inhibition of TLR2/MyD88/NF‐κB pathway	Not a herbal oral intervention (e.g., collagen/HA/animal‐derived or synthetic ingredients)
25	Dusabimana T.	2024	Oyster hydrolysate ameliorates UVB‐induced skin dehydration and barrier dysfunction.	Not a herbal oral intervention (e.g., collagen/HA/animal‐derived or synthetic ingredients)
26	Klinngam W	2024	Skin rejuvenation efficacy and safety evaluation of *Kaempferia parviflora* standardized extract (BG100) in Human 3D skin models and clinical trial.	In vitro skin models
27	Morakul	2024	The evidence from in vitro primary fibroblasts and a randomized, double‐blind, placebo‐controlled clinical trial of tuna collagen peptides intake on skin health.	In vitro and not a herbal oral intervention (e.g., collagen/HA/animal‐derived or synthetic ingredients)
28	Putthong C.	2024	Efficacy of natural β‐carotene chewable tablets derived from banana (Musa AA) pulp in reducing UV‐induced skin erythema.	Non‐oral intervention or insufficient evidence of oral supplement
29	Seong S.H.	2024	Low‐molecular‐weight collagen peptides supplement promotes a healthy skin: A randomized, double‐blinded, placebo‐controlled study.	Not a herbal oral intervention (e.g., collagen/HA/animal‐derived or synthetic ingredients)
30	Seong S.H.	2024	Oral consumption of Bonito fish‐derived elastin peptide (VGPG Elastin) improves biophysical properties in aging skin: A randomized, double‐blinded, placebo‐controlled study.	Not a herbal oral intervention (e.g., collagen/HA/animal‐derived or synthetic ingredients)
31	Sung H.K.	2024	Anti‐wrinkle and skin moisture efficacy of 7‐MEGA: A randomized, double‐blind, placebo comparative clinical trial.	Not a herbal oral intervention (e.g., collagen/HA/animal‐derived or synthetic ingredients)
32	Vleminckx S.	2024	Influence of collagen peptide supplementation on visible signs of skin and nail health and ‐aging in an East Asian population: A double blind, randomized, placebo‐controlled trial.	Not a herbal oral intervention (e.g., collagen/HA/animal‐derived or synthetic ingredients)
33	Wang Y.	2024	Combined microfocused ultrasound and delicate pulsed light for facial rejuvenation: A prospective, randomized, and split‐face study.	Not a herbal oral intervention (e.g., collagen/HA/animal‐derived or synthetic ingredients)
34	Zhang L.	2024	Dual intervention on the gut and skin microbiota attenuates facial cutaneous aging.	Not a herbal oral intervention (e.g., collagen/HA/animal‐derived or synthetic ingredients)
35	Žmitek K.	2024	The effects of dietary supplementation with collagen and vitamin c and their combination with hyaluronic acid on skin density, texture and other parameters: A randomized, double‐blind, placebo‐controlled trial.	Not a herbal oral intervention (e.g., collagen/HA/animal‐derived or synthetic ingredients)
36	Bai X.D.	2025	Clinical trial of salmon nasal cartilage‐derived proteoglycans on human facial antiaging: A randomized, double‐blind, placebo‐controlled study.	Not a herbal oral intervention (e.g., collagen/HA/animal‐derived or synthetic ingredients)
37	He Y.	2025	Multi‐plant concentrated powder improved skin whitening: A double‐blinded, randomized, and placebo‐controlled clinical study.	Not a herbal oral intervention (e.g., collagen/HA/animal‐derived or synthetic ingredients)
38	Kim M.	2025	Effects of hawthorn fruit supplementation on facial skin phenotypes and leukocyte telomere length stratified by TERT polymorphisms.	No instrumental skin aging outcome; does not meet predefined outcome criteria
39	Michelini S.	2025	Non‐invasive imaging for the evaluation of a new oral supplement in skin aging: A case‐controlled study.	Not a herbal oral intervention (e.g., collagen/HA/animal‐derived or synthetic ingredients)
40	Mohamadi	2025	Comparative analysis of erbium: Glass 1550 nm and combined erbium: YAG & Nd: YAG lasers for perioral rejuvenation: A prospective study.	Not a herbal oral intervention
41	Navarro P.	2025	Skin photoprotection and anti‐aging benefits of a combination of rosemary and grapefruit extracts: Evidence from in vitro models and human study.	Not a herbal oral intervention (e.g., collagen/HA/animal‐derived or synthetic ingredients), not RCT
42	Tursi F.	2025	The effects of an oral supplementation of a natural keratin hydrolysate on skin aging: A randomized, double‐blind, placebo‐controlled clinical study in healthy women.	Not a herbal oral intervention (e.g., collagen/HA/animal‐derived or synthetic ingredients)
43	Zhao M.	2025	Efficacy and mechanism of Bazi Bushen capsule on skin laxity: A combination of clinical and network pharmacology study.	Not a RCT
44	da Silva Junior S.V.	2025	Comparing ready‐to‐use and powder abobotulinumtoxina for glabellar lines: A randomized, controlled, triple‐blinded clinical trial.	Not a herbal oral intervention (e.g., collagen/HA/animal‐derived or synthetic ingredients)

A comprehensive literature search was performed in the Cochrane Library, PubMed, and ScienceDirect on August 8, 2025. Controlled vocabulary and keyword combinations relating to *oral supplementation*, *herbal products*, and *skin aging/aging* were used. Search parameters were tailored to each database: the Cochrane Library search was restricted to intervention reviews and trials published from January 1, 2020, to August 8, 2025; the PubMed search applied filters for clinical trial, randomized controlled trial (RCT), humans, English, and the past 5 years; and the ScienceDirect search was limited to research articles published in 2020–2025, within Medicine and Dentistry, in English, with open‐access or open‐archive availability.

The inclusion criteria for this systematic review encompassed clinical trials involving human subjects over 20 years old who exhibited visible signs of skin aging, such as wrinkles, loss of elasticity, or uneven pigmentation. Only studies examining interventions that used oral herbal supplementation as the sole treatment were included. Additionally, eligible studies had to be published between January 2020 and August 2025 and be written in English. Exclusion criteria comprised studies that employed combined treatment approaches, such as oral supplementation alongside topical therapies. Trials involving participants with underlying skin conditions, as well as studies conducted on animals, in vitro experiments, and non‐clinical review articles, were also excluded.

This review included only clinical trials published between 2020 and 2025, reflecting recent methodological advances in dermatology and nutrition research. Recent RCTs increasingly apply standardized, validated measures, yielding more reliable outcomes. Bibliometric data also show that most high‐impact antiaging studies emerged within this period, ensuring the synthesis focuses on the most current and clinically relevant evidence.

The initial screening was conducted based on titles and abstracts, followed by a full‐text review. Two reviewers independently assessed studies for eligibility. Disagreements were resolved through discussion with a third reviewer. When there was a disagreement between reviewers, the next reviewer decided on the inclusion or exclusion of the study. Risk of bias for RCTs was assessed using the Cochrane RoB 2 tool, considering five domains: (1) bias arising from the randomization process; (2) bias due to deviations from intended interventions (effect of assignment); (3) bias due to missing outcome data; (4) bias in measurement of the outcome; and (5) bias in selection of the reported result. For each study and domain, we rated risk of bias as “low risk,” “some concerns,” or “high risk,” followed by an overall judgment. The relevant findings of each selected study, including the first author, year of publication, sample size, study type, intervention (oral herbal), comparison (placebo or standard treatments), and results, were extracted from the original studies. A predefined protocol guided the review's objectives, eligibility criteria, outcomes, and methods. The protocol was not registered in PROSPERO; however, the review was conducted systematically using predefined eligibility criteria and focused exclusively on evidence derived from included clinical trials.

## 3 Result and Discussion

**FIGURE 2:**
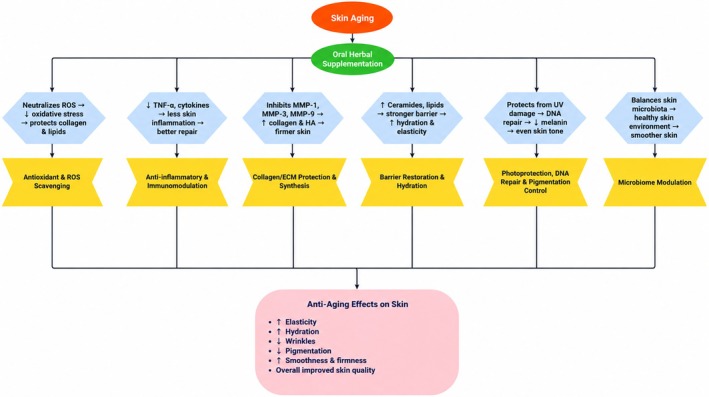
Schematic overview of oral herbal supplementation and their main antiaging mechanisms.

From 2856 screened studies, 12 high‐quality RCTs (2020–2025) were included after rigorous screening and Cochrane RoB2 assessment (Tables 3 and 4). Overall, the findings indicate that oral herbal supplements improve skin elasticity, hydration, wrinkles, and oxidative stress by antioxidant, anti‐inflammatory, and matrix metalloproteinase (MMP) inhibitory mechanisms, as well as by enhancing collagen and hyaluronic acid synthesis, lipid balance, hormonal regulation, and microbiome modulation, thereby strengthening skin firmness, smoothness, hydration, and environmental resilience (Table 5 and Figure 2).

**TABLE 3:** Oral herbal supplementation on skin aging: human studies.

No	First author	Year	Population	Methods	Intervention	Outcome
1	Da‐Bin Myung [11]	2020	Healthy males/females (35–60 y), *n* = 151, wrinkles grade > 3.	Randomized, double‐blind, placebo‐controlled study. Skin parameter evaluation using visual assessment by dermatologist, 3D skin analyzer, Corneometer, Tewameter, and Cutometer.	*Hydrangea serrata* 300 mg or 600 mg/day, 12 weeks.	Improved elasticity, reduced roughness and texture changes; no adverse effects.
2	Pakagamon Tumsutti [12]	2021	Menopausal women aged 45–60 with facial fine lines/wrinkles (Glogau II–III).	RCT, double‐blind, placebo‐controlled, 12 weeks. Skin parameter evaluation using visual assessment by dermatologist, Cutometer, Mexameter, Glossymeter, Corneometer, Tewameter, and Artificial intelligence skin analyzer. Biochemical assessments using blood sample to analyzed GSH and MDA activity.	Estosalus (*Glycine max, Cimicifuga racemosa, Vitex agnus‐castus, Oenothera biennis*), 1 capsule before breakfast daily.	Significant improvements in skin elasticity, roughness, smoothnes, scaliness, wrinkles; ↑GSH, and ↓MDA.
3	Hua Zhao [13]	2021	Healthy Chinese adults (18–60 years). Mean age of 41.2 (standard deviation ± 10.8) years.	Randomized, double‐blind, placebo‐controlled, crossover study. Skin parameter evaluation using Tewameter, Corneometer, Cutometer, and Skin colorimeter.	Pycnogenol 100 mg/day (2 × 50 mg), 12 weeks.	Improved skin hydration, reduced TEWL, prevented skin darkening, enhanced elasticity.
4	Tsung‐Yi Tsai [14]	2021	Healthy adults, mean age 41.2 years (*n* = 76).	Monocentre, double‐blind, randomized, placebo‐controlled, crossover study. Skin parameter evaluation using Corneometer, Chroma Meter, Artificial Intelligence skin analyzer, and DermaLab ultrasound.	50 mL of djulis for 8 weeks once daily.	Improvements in skin moisture, brightness, elasticity, crow's feet, texture, wrinkles, pores, and collagen content.
5	Young‐Min Ham [15]	2022	Women aged 40–60 with dry skin (< 48 Arbitary Units) and periorbital wrinkles.	RCT, double‐blind, placebo‐controlled study. Skin parameter evaluation using corneometer, 3D skin analyzer, Artificial Intelligence skin analyzer, Corneometer, Tewameter, Cutometer, and Spectrophotometer.	Green mandarin extract, 300 mg/day for 12 weeks.	↓Wrinkle depth, volume, roughness; no adverse events.
6	Vincenzo Nobile [16]	2022	Healthy adults (35–55 years), mild‐moderate aging signs.	Multicenter, stratified, randomized, double‐blind, placebo‐controlled study. Skin parameter evaluation using Corneometer, and Cutometer. Total skin antioxidant capacity measured using a ferric reducing antioxidant power (FRAP) assay, melanin intensity and skin radiance using colorimetric method, and skin rlipoperoxide using MDA assay, and wrinkles using 3D scanner.	Red Orange Complex H 100 mg/day, 56 days.	Improved photoprotection, antioxidant status, moisturization, elasticity, reduced TEWL, melanin intensity, and wrinkles.
7	Chen‐Meng Kuan [17]	2022	Healthy adults over 20 years of age.	Randomized, double‐blind, parallel, and placebo‐controlled study. Skin parameter evaluation using artificial intelligence skin analyzer system, Chroma meter, and Corneometer.	*Crassocephalum rabens* capsule (180 mg/day), ever day for 4 weeks.	Reduced wrinkles and improved skin elasticity, collagen content, brightness, and hydration.
8	Catherine Kern [18]	2023	72 healthy Caucasian females aged 30–60 years old with dry skin and mild to moderate signs of chronological or photo aging	Randomized, double‐blind, and placebo controlled study. Skin parameter evaluation instrument using Corneometer, Tewameter, Cutometer, and 3D scanner. Lipidomic analysis performed using tape stripping and LIPID MAPS system.	Wheat polar lipid complex in oil or powder form (WPLC‐O 70 mg/day and WPLC‐P 30 mg/day, respectively), two capsules everyday for 56 days.	Increased in skin hydration, elasticity, and smoothness while decreasing TEWL, roughness, and wrinkle depth.
9	Elizabeth Tarshish [19]	2023	59 healthy women aged 35–55 with sensitive facial skin.	RCT, double‐blind, placebo‐controlled study. Skin parameter evaluation using Tewameter and Cutometer.	Lumenato capsule (yellow tomato carotenoids), 10 mg/day total carotenoids for 12 weeks.	↑Skin barrier strength, ↑firmness and elasticity, and ↓TEWL.
10	Lindsey Christman [20]	2024	Women aged 25–65 (Fitzpatrick Type II–III), 12 per age group (25–39 and 40–65).	RCT, double‐blind, placebo‐controlled, crossover study. Skin parameter evaluation using Cutometer, Visioscan, Tewameter, Corneometer, Skin pH meter probe, Skin Colorimeter, and Mexameter. Superoxide dismutase (SOD) and gluthatione perocidase (PDx) enzymes were measured through colorimetric assays. TNF‐α were measured by Enzyme‐linked immunosorbent assay (ELISA). Skin lipids were sampled by sequential tape stripping on the forearm. Cutaneous microbiota sample were obtained by swabbing the forearm with DNA‐free sterile cotton‐tipped swabs, stored at −80°C and sent to Cosmo ID for analysis.	Cranberry beverage: 192.9 mg procyanidins, 19.5 mg anthocyanins, 24.2 mg flavonols per bottle, daily for 6 weeks.	Improved skin elasticity/smoothness (esp. > 40 years), ↑SOD, ↓TNF‐α, modulated microbiome/lipids.
11	Vincenzo Nobile [21]	2024	66 caucasian women aged 35–65, with crow's feet and mild skin slackness.	Skin antioxidant capacity was measured on skin samples taken with foils and undergone a ferric reducing antioxidant power (FRAP) assay.	Fermented bilberry extract (Sepitone), 100 mg/day for 12 weeks.	↓Wrinkle depth 10.6%, ↑firmness 13.3%, elasticity 12.4%, skin tone, antioxidant capacity ↑20.8%.
12	Daisuke Aoyagi [22]	2025	Healthy Japanese women (40–50 y), *n* = 48.	Randomized, double‐blind, placebo‐controlled trial. Skin parameter evaluation using Corneometer, Tewameter, Mexameter, colorimeter, Cutometer, image analysis application, Artifial intelligence skin analyzer, Sebumeter, noncontact radiation thermometer and skin‐pH‐meter.	Blue Rose Extract 100 mg/day for 4 weeks.	Improved water content also reduced melanin index, stains, wrinkles, and roughness.

**TABLE 4:** Risk of bias (RoB2) assessment for included randomized controlled trials.

No.	First author, year	Domain 1: randomization process	Domain 2: deviations from intended interventions	Domain 3: missing outcome data	Domain 4: measurement of outcomes	Domain 5: selection of reported result	Overall RoB
1	Myung, 2020	Some concerns	Low risk	Low risk	Low risk	Low risk	Some concerns
2	Tumsutti, 2022	Some concerns	Low risk	Low risk	Low risk	Some concerns	Some concerns
3	Zhao, 2021	Some concerns	Low risk	Low risk	Low risk	Some concerns	Some concerns
4	Tsai, 2022	Some concerns	Low risk	Low risk	Low risk	Some concerns	Some concerns
5	Ham, 2022	Low risk	Low risk	Low risk	Low risk	Low risk	Low risk
6	Nobile, 2022	Low risk	Low risk	Low risk	Low risk	Some concerns	Som concerns
7	Kuan, 2022	Some concerns	Low risk	Low risk	Low risk	Low risk	Some concerns
8	Kern, 2024	Some concerns	Low risk	Low risk	Low risk	Some concerns	Some concerns
9	Tarshish, 2023	Some concerns	Low risk	Low risk	Low risk	Low risk	Some concerns
10	Christman, 2024	Some concerns	Low risk	Low risk	Low risk	Low risk	Some concerns
11	Nobile, 2024	Low risk	Low risk	Low risk	Low risk	Some concerns	Some concerns
12	Aoyagi, 2025	Some concerns	Low risk	Low risk	Low risk	Low risk	Some concerns

**TABLE 5:** Categorization of herbal compounds by antiaging mechanisms.

No	Main mechanism	Oral herbal
1	Antioxidant and reactive oxygen species (ROS) scavenging	*hydrangea serrata* [11], estosalus [12], pycnogenol [13], djulis [14], green mandarin extract [15], *crassocephalum rabens* [17], lumenato [19], cranberry beverage [20], fermented bilberry extract [21], blue rose extract [22]
2	Anti‐inflammatory and immunomodulation	Young‐Min Ham [15], *Crassocephalum rabens* [17], cranberry beverage [20], fermented bilberry extract [21], blue rose extract [22]
3	Collagen/extracellular matrix (ECM) protection and synthesis	*Hydrangea serrata* [11], Djulis [14], green mandarin extract [15], red orang complex H [16], fermented bilberry extract [21], *Crassocephalum rabens* [17]
4	Barrier restoration and hydration	*Hydrangea serrata* [11], pycnogenol [13], wheat polar lipid complex [18], lumenato [19], Blue rose extract [22]
5	Photoprotection, DNA repair, and pogmentation control	Red orang complex H [16], lumenato [19], Blue rose extract [22]
6	Microbiome modulation	Cranberry beverage [20]

Across the 12 included trials, elasticity was the most consistently improved outcome, reported in 10 of 12 studies regardless of supplement class, dose, or duration [11, 12, 13, 14, 15, 16, 17, 18, 19, 20, 21, 22]. This near‐universal finding likely reflects the sensitivity of Cutometer‐based measurement rather than a convergent mechanism. Elasticity improvement is a functional endpoint that multiple distinct pathways (antioxidant, hormonal, barrier lipid, and collagen‐directed) can all plausibly influence, making it a poor discriminator between mechanistic classes [8, 9, 10].

Hydration was the second most consistent outcome (9 of 12 studies), but with an important distinction that not all hydration improvements are mechanistically equivalent. Wheat polar lipid complex (WPLC), Pycnogenol, and *Hydrangea serrata* improved both hydration and TEWL simultaneously, indicating genuine barrier restoration through ceramide replenishment or hyaluronic acid synthesis [11, 13, 18, 23, 24, 25, 26]. By contrast, Djulis and *Crassocephalum rabens* improved hydration without measurable TEWL reduction, suggesting surface‐layer water retention rather than structural barrier repair [14, 17].

TEWL reduction as the most specific marker of epidermal barrier function was achieved by only five interventions such as Pycnogenol, Red Orange Complex H, *Hydrangea serrata*, WPLC, and Lumenato [11, 13, 16, 18, 19]. These five share either lipid‐modulating activity or demonstrated hyaluronic acid synthase influence, reinforcing that TEWL improvement requires a mechanistic action on barrier architecture, not simply antioxidant load. The remaining seven interventions, despite producing elasticity and hydration gains, failed to move TEWL, which limits their interpretation as true barrier‐restoring agents [23, 24, 25, 26, 27].

Wrinkle reduction showed the greatest variability. Green mandarin and fermented bilberry produced the most specific wrinkle outcomes with depth and volume reductions without broad hydration effects consistent with a collagen or MMP directed mechanism [15, 21, 28, 29]. Djulis and Red Orange Complex *H* also reduced wrinkles but alongside multiple other outcomes, making the mechanistic attribution less interpretable [14, 16, 30, 31]. Blue Rose extract and *C. rabens* showed wrinkle improvements at only 4 weeks implicating acute anti‐inflammatory or surface‐level antioxidant activity [17, 22, 32, 33, 34, 35].

Two findings stand apart from the general pattern. Estosalus is the only intervention that provided direct biochemical corroboration of its proposed mechanism within the trial itself for the measurable Glutathione (GSH) increases and malondialdehyde (MDA) decreases alongside clinical improvements making it the strongest mechanistic case in this review [12, 36, 37]. Particularly, for postmenopausal populations where hormonal antioxidant depletion is the primary driver of skin aging. The cranberry beverage is the only intervention whose benefit was age‐stratified and microbiome‐associated suggesting an indirect gut‐skin immune axis mechanism that is categorically different from the direct cutaneous antioxidant activity claimed by all other included supplements [20, 38, 39]. These two studies are therefore not directly comparable to the rest of the evidence base and warrant separate consideration in future research.

Across the 12 included trials, skin elasticity and hydration emerged as the most consistently improved outcomes, reported in at least nine studies spanning diverse herbal interventions, including Pycnogenol, Hydrangea serrata, WPLC, fermented bilberry, and Djulis. Wrinkle depth reduction was also commonly reported, though the magnitude of effect varied substantially across studies, reflecting differences in study duration, population characteristics, dosing regimens, and outcome measurement tools. A summary table categorizing outcomes into standardized domains could be seen on Table 6.

**TABLE 6:** Outcome summary per category.

No	Outcomes	First author
1	Improved hydration	Da‐Bin Myung [11], Pakagamon Tumsutti [12], Hua Zhao [13], Tsung‐Yi Tsai [14] Vincenzo Nobile [16], Chen‐Meng Kuan [17], Catherine Kern [18], Elizabeth Tarshish [19], Daisuke Aoyagi [22]
2	Improved elasticity	Da‐Bin Myung [11], Pakagamon Tumsutti [12], Hua Zhao [13], Tsung‐Yi Tsai [14], Vincenzo Nobile [16], Chen‐Meng Kuan [17], Catherine Kern [18], Elizabeth Tarshish [19], Lindsey Christman [20], Vincenzo Nobile [21], Daisuke Aoyagi [22]
3	Reduced TEWL	Da‐Bin Myung [11], Hua Zhao [13], Vincenzo Nobile [16], Catherine Kern [18], Elizabeth Tarshish [19]
4	Wrinkle reduction	Da‐Bin Myung [11], Pakagamon Tumsutti [12], Tsung‐Yi Tsai [14], Young‐Min Ham [15], Vincenzo Nobile [16], Chen‐Meng Kuan [17], Catherine Kern [18], Vincenzo Nobile [21], Daisuke Aoyagi [22]
5	Increased skin smoothness	Da‐Bin Myung [11], Pakagamon Tumsutti [12], Catherine Kern [18], Lindsey Christman [20]
6	Improved skin texture	Da‐Bin Myung [11], Pakagamon Tumsutti [12], Young‐Min Ham [15], Catherine Kern [18], Daisuke Aoyagi [22]
7	Reduced skin darkening/increased brightness	Hua Zhao [13], Tsung‐Yi Tsai [14], Chen‐Meng Kuan [17]
8	Reduced crow's feet	Tsung‐Yi Tsai [14]
9	Increased collagen content	Tsung‐Yi Tsai [14], hen‐Meng Kuan [17]
10	Increased photoprotection	Vincenzo Nobile [16]
11	Reduced UV spots	Chen‐Meng Kuan [17]
12	Reduced melanin intensity	Vincenzo Nobile [16], Daisuke Aoyagi [22]
13	Increased skin firmness and barrier	Elizabeth Tarshish [19], Vincenzo Nobile [21]
14	Reduced wrinkle depth	Young‐Min Ham [15], Vincenzo Nobile [21]

Comparison across interventions reveals clinically meaningful distinctions. Multi‐ingredient formulations, such as Estosalus, demonstrated broader benefits in postmenopausal women by concurrently targeting hormonal, antioxidant, and barrier repair pathways—an advantage attributable to the combined estrogenic activity of *Glycine max* isoflavones and the adaptogenic properties of *Vitex agnus‐castus* . In contrast, single‐extract preparations such as Pycnogenol and fermented bilberry extract demonstrated robust improvements in hydration and elasticity among general adult populations, with the latter achieving a notable 10.6% reduction in wrinkle depth and a 20.8% increase in antioxidant capacity after 12 weeks. Notably, TEWL reduction—a validated marker of epidermal barrier function—was more consistently associated with lipid‐rich interventions (WPLC, Pycnogenol) than with flavonoid‐dominant extracts, suggesting mechanistically distinct pathways: lipid‐based supplements appear to act primarily through ceramide replenishment and stratum corneum restoration, whereas polyphenol‐rich extracts exert effects predominantly via antioxidant and anti‐inflammatory signaling. Regarding mechanistic claims, several proposed biological explanations—including collagen synthesis stimulation via the transforming growth factor beta (TGF‐β)/supressor of mothers against decapentaplegic 3 (Smad3) pathway, MMP‐1/MMP‐3 inhibition, and microbiome remodeling—are supported primarily by preclinical or indirect evidence and should be considered working hypotheses rather than established mechanisms in humans. Direct clinical evidence linking these molecular events to observed skin changes remains limited; future trials incorporating biopsy‐based or biomarker‐confirmed mechanistic endpoints are needed to substantiate these pathways. The microbiome‐modulating effect of the cranberry polyphenol beverage, evidenced by changes in skin‐associated microbial communities alongside reductions in tumor necrosis factor alpha (TNF‐α), represents a, particularly, novel and understudied avenue warranting dedicated mechanistic investigation.

Several methodological limitations of this review require explicit emphasis. First, restricting inclusion to open‐access publications, while a deliberate decision to ensure full‐text verifiability and transparency, represents a critical constraint that may have substantially compromised comprehensiveness. Risk of bias assessment revealed that 11 of 12 included RCTs (92%) were rated as having “some concerns” overall under the Cochrane RoB 2 tool (Table 4). The predominant sources of concern were incomplete reporting of randomization procedures (Domain 1: 9 of 12 studies) and potential selective outcome reporting (Domain 5: 6 of 12 studies). Although no study was rated as high risk, the pervasive “some concerns” classification meaningfully limits the certainty of conclusions. Specifically, inadequate randomization reporting raises the possibility that allocation concealment was insufficient in certain trials, potentially inflating treatment effect estimates, while selective outcome reporting introduces the risk that only statistically favorable outcomes were disseminated. Readers are therefore cautioned against interpreting the consistently positive direction of findings as confirmation of definitive efficacy; these results should be considered exploratory and hypothesis‐generating, requiring corroboration from methodologically rigorous, prospectively registered trials with pre‐specified outcomes.

The safety profile of oral herbal supplements across the included trials was generally favorable, with only mild, transient adverse effects primarily gastrointestinal discomfort and headache reported at frequencies comparable between intervention and control groups, and no serious product‐related events documented. However, this reassuring profile must be contextualized against an important caveat: the majority of included trials were short‐term, with durations ranging from only 4 to 12 weeks. Such brief follow‐up periods are fundamentally insufficient to detect rare adverse events, cumulative toxicities, or late‐onset effects that may emerge with prolonged use. The apparent tolerability of these supplements in short‐duration RCTs should not be extrapolated to long‐term safety in clinical practice. Postmarket pharmacovigilance, longer‐duration trials, and standardized adverse event reporting frameworks are urgently needed before definitive clinical recommendations can be issued.

## Author Contributions

Gisela Meydilin Sanjaya and Brilian Veda Kartika Putri conducted the initial screening of titles and abstracts and performed the full‐text review. Both reviewers independently assessed studies for eligibility. Any disagreements were discussed with the third reviewers (Hany Anneke, Nur Atik, and Nayla Majeda Alfarafisa) to reach a consensus. In cases where disagreement persisted, Astrid Feinisa Khairani made the final decision regarding the inclusion or exclusion of studies.

## Funding

This work was supported by Universitas Padjadjaran, PUPA.

## Ethics Statement

This study is a systematic review that synthesized data from previously published research. Therefore, no new human or animal subjects were involved, and ethical approval or informed consent was not required. All data analyzed in this review had already received ethical clearance from their respective institutional review boards.

## Conflicts of Interest

The authors declare no conflicts of interest.

## Data Availability

The data that support the findings of this study are available from the corresponding author upon reasonable request.
